# Barriers and Facilitators to HIV Pre-Exposure Prophylaxis Uptake among Men Who Have Sex with Men Who Use Stimulants: A Qualitative Study

**DOI:** 10.1007/s10461-022-03633-5

**Published:** 2022-03-18

**Authors:** Adam Viera, Jacob J. van den Berg, Collette D. Sosnowy, Nikita A. Mehta, E. Jennifer Edelman, Trace Kershaw, Philip A. Chan

**Affiliations:** a Department of Social and Behavioral Sciences, Yale School of Public Health, 60 College Street, New Haven, CT 06510; b Center for Interdisciplinary Research on AIDS, 135 College Street, New Haven, CT 06510; c Warren Alpert Medical School of Brown University, 222 Richmond St, Providence, RI 02903; d Department of Behavioral and Social Sciences, Brown University School of Public Health, 121 S Main St, Providence, RI 02903; e Providence/Boston Center for AIDS Research, 164 Summit Avenue CFAR Building, Room 134 Providence, RI 02906; f Department of Epidemiology, Harvard T.H. Chan School of Public Health, 677 Huntington Avenue, Boston, MA 02115; g Department of Internal Medicine, Yale School of Medicine, 367 Cedar St, New Haven, CT 06510

## Abstract

The HIV epidemic disproportionately impacts men who have sex with men (MSM), particularly those who use stimulants. We explored barriers and facilitators to pre-exposure prophylaxis (PrEP) uptake among this population. From June 2018 through February 2019, we conducted semi-structured interviews in Providence, Rhode Island, and New Haven, Connecticut, with 21 MSM who reported recent (past six months) stimulant use. We identified individual, interpersonal, and structural barriers to PrEP, including: 1) high awareness but mixed knowledge of PrEP, resulting in concerns about side effects and drug interactions; 2) interest that was partly determined by substance use and perceived HIV risk; 3) fragmented and constrained social networks not conducive to disseminating PrEP information; and 4) PrEP access, such as insurance coverage and cost. Our findings suggest potential approaches to increase PrEP uptake in this group, including promotion through mainstream and social media, clarifying misinformation, and facilitating increased access through structural interventions.

## RESUMEN

La epidemia del VIH afecta de manera desproporcionada a los hombres que tienen sexo con hombres (HSH), particularmente a aquellos que usan estimulantes. Exploramos las barreras y los facilitadores para la adopción de la profilaxis previa a la exposición (PrEP) en esta población. Desde junio de 2018 hasta febrero de 2019, realizamos entrevistas semiestructuradas en Providence, Rhode Island y New Haven, Connecticut, con 21 HSH que nos informaron de haber usado estimulantes recientemente (en los últimos seis meses). Identificamos barreras individuales, interpersonales, y estructurales para la PrEP, que incluyen: 1) gran conciencia pero conocimiento mixto de la PrEP, lo que produce preocupaciones sobre los efectos secundarios y las interacciones farmacológicas; 2) interés que fue determinado en parte por el uso de sustancias y el riesgo percibido de VIH; 3) redes sociales fragmentadas y restringidas que no conducen a la difusión de información sobre la PrEP; y 4) problemas con el acceso a la PrEP, como la cobertura y el costo del seguro. Nuestros hallazgos sugieren enfoques potenciales para aumentar la aceptación de la PrEP en esta población, incluida la promoción a través de los medios tradicionales y sociales, aclarando la información errónea, y facilitando un mayor acceso a través de intervenciones estructurales.

## INTRODUCTION

Substance use (use of alcohol, tobacco, or other drugs) is highly prevalent among gay, bisexual, and other men who have sex with men (MSM) and significantly elevates risk of HIV infection (1–4). Stimulant use (use of cocaine, amphetamines, methamphetamine or prescription stimulants outside of as directed by a medical professional), in particular, is twice as likely to be reported by MSM compared to other men (5, 6). Stimulant use is increasingly a public health concern throughout the United States (7–11), with rates of drug overdose involving stimulants increasing across the country (12, 13). Moreover, stimulant use is associated with a greater likelihood of condomless anal sex, sex with an HIV-positive partner, and other behaviors associated with HIV acquisition (14–24).

Pre-exposure prophylaxis (PrEP) is a highly effective HIV prevention method and has demonstrably reduced HIV infections among MSM (25). While PrEP use has increased among MSM in the United States (26), rates of uptake continue to be slow (27), with under 9% of those “at substantial risk of HIV infection” actively taking PrEP and stark disparities by race/ethnicity and insurance status persisting (28). Barriers include low PrEP awareness and knowledge, low perceived HIV risk, high cost, fear of stigma (e.g., being seen as promiscuous or HIV-positive), concerns about side effects, and perceived burden of taking a pill every day (29–33). In addition, structural barriers to PrEP uptake include lack of access to PrEP care (e.g., due to location of provider, cost, availability), language barriers, insurance coverage, and bias among medical providers (29, 34–37).

Rates of PrEP uptake have remained especially low among MSM who use stimulants (38–40). Research shows that scaling up PrEP among MSM who use stimulants could reduce new HIV infections by 19% over ten years (41). Moreover, while evidence for the effect of substance use on PrEP non-adherence and discontinuation is mixed (42–50), some studies have connected stimulant use to increased risk of acquiring HIV and decreased PrEP effectiveness (51, 52). The iPrEx study investigated this relationship and found no association between substance use and PrEP retention in care (53); however, a PrEP acceptability study surveying MSM in Boston who use stimulants found that 40% believed their substance use would adversely affect their adherence, a significantly higher percentage compared to MSM who use alcohol (54). To our knowledge, no studies have qualitatively explored the barriers and facilitators to PrEP uptake among MSM who use stimulants.

One potential approach to improving PrEP uptake among MSM who use stimulants includes leveraging social networks to find and engage this group. Interventions that leverage social networks have previously demonstrated success in engaging both MSM and people who use drugs in reducing HIV risk behaviors, such as condom use and safer injection practices (55–59). Researchers have explored how social networks can be used to promote PrEP awareness and uptake, mostly among MSM (60–62); however, studies examining how to leverage the social networks of MSM who use stimulants to promote PrEP uptake are lacking. Our previous research demonstrated that PrEP clinics have not been effective in reaching MSM who use stimulants (38). The goal of our current study, Connect-2-PrEP, was to characterize barriers and facilitators related to PrEP uptake among MSM who use stimulants and to explore the potential for social networks to serve as a point of intervention.

## METHODS

### Participants and Setting

Participants were recruited in New Haven, Connecticut and Providence, Rhode Island, two small cities in the northeastern United States. Participants were recruited through partnering community-based organizations, providing primarily HIV prevention services to MSM or LGBTQ communities more broadly, as well as online advertisements on Craigslist. All participants were screened prior to the interview to determine whether they met the following eligibility criteria: 1) were 18 years of age or older, 2) spoke English or Spanish, 3) identified as cisgender male, 4) reported lifetime oral or anal sex with another man, and 5) reported stimulant use in the past six months. HIV serostatus was not established as criteria for inclusion or exclusion given how HIV-positive MSM were likely to be in social networks with HIV-negative MSM and would provide important perspectives around HIV prevention among MSM who use stimulants.

### Data Collection

Four members of the research team (AV, CDS, JJVDB, NAM) conducted semi-structured individual in-depth interviews. Interviews were conducted from June 2018 through February 2019. Only research staff and the participant were present for the interview. Research staff explained the purpose of the study and obtained informed consent from eligible participants prior to the interview. Interviews lasted approximately 60 minutes and were all conducted in English.

The interview guide was developed by the research team, reviewed by community partners, and pilot tested among the research team before use. The interview guide was developed following principles of grounded theory (63, 64) and sought to understand behavioral, social, and structural factors that may impact PrEP uptake for MSM who use stimulants. The interview guide included questions about PrEP awareness and knowledge (followed by a definition and description of PrEP), PrEP attitudes and experiences; sexual behaviors; drug use behaviors; social networks; and recommendations for improving PrEP uptake. The interview guide is available as supplementary material **(see Appendix A)**.

After the interview, participants completed a brief survey to assess demographic information HIV risk behaviors (including incarceration history), and substance use, the latter of which was assessed via the Alcohol Use Disorders Identification Test—Consumption (AUDIT-C) (65) and the Alcohol, Smoking and Substance Involvement Screening Tool - Lite (ASSIST-Lite) (66). Each participant received a $50 gift card. All research activities were approved by the Institutional Review Boards at Yale University and The Miriam Hospital.

We intended to recruit 30 participants, but stopped recruitment before this target had been reached. We determined we had reached saturation through ongoing discussions of interviews. This is consistent with research showing that saturation is reached with about 12 interviews (67, 68).

### Data Analysis

Interviews were digitally audio-recorded and transcribed by an outside HIPAA-certified transcription company and reviewed for accuracy by research staff. Transcripts were uploaded to Dedoose for coding and analysis (69). Thematic analysis was employed for this process (70). The research staff who conducted the interviews coded the first two transcripts and met to resolve discrepancies and refine the coding scheme. Research staff repeated this process until consistent consensus was achieved, then coded the remaining transcripts, with at least two members of the research team coding each transcript. After coding, the research team inductively identified key themes related to PrEP attitudes as well as barriers and facilitators to PrEP uptake among participants. Using a thematic matrix, the research team categorized themes according to the factors of the social ecological model (71), which we selected given its widespread use in intervention design. For quantitative survey data, we ran descriptive statistics using R version 3.6.0 (72) via RStudio (73).

### Positionality Statement

All interviewers and coders were trained and/or experienced in qualitative research methodology. Author 1 is a Latinx and White gay male who was a doctoral candidate at the time of conducting the interviews. He has a long history of working in the fields of HIV prevention and substance use. Author 2 is a White gay male who was Assistant Professor at the time of the interviews. He has worked on numerous HIV prevention studies with MSM who use substances. Author 3 is a White female who was an Assistant Professor at the time of the interviews. She has worked on numerous projects related to HIV prevention, primarily access to PrEP for high-risk populations. Author 4 is an Asian-Indian female who was a Master of Public Health candidate concentrating in social and behavioral sciences at the time of conducting the interviews. She has a history of working with PrEP education and HIV prevention. The authors, who were all fully employed in or affiliated with academic institutions at the time of the interviews, bring their own personal experiences and understanding of sexual minority males, stimulant use, and HIV, which may affect their interpretation of the data.

## RESULTS

### Overall Sample

We conducted 24 interviews (18 in Providence,RI and 6 in New Haven, CT). Three interviews were removed from analysis when further review of the transcripts revealed these participants did not meet all eligibility criteria despite initial screening (i.e., two participants did not report any sexual activity with another man and one participant did not identify as cisgender male). Two participants reported recently starting PrEP while another participant reported having taken PrEP in the past.

### Participant Characteristics

Among the 21 participants included in analysis, most identified as White (57.1%) and gay (52.4%). The majority of participants reported being HIV-negative (76.2%). Nearly two-thirds had been previously incarcerated (61.9%) (Table 1).

Participants reported an average of four anal sex partners, four vaginal sex partners, four female oral sex partners, and nine male oral sex partners in the past three months. Over half of participants (57.1%) exchanged sex for drugs, money, or something else in the past three months. Nearly half of participants (45.5%) had ever had a sexually transmitted disease and over a third (36.4%) had been sexually assaulted at some point in their lifetime. Most participants reported meeting sexual partners at bars (45.5%) or online (45.5%) Nearly half of participants (40.9%) had ever injected drugs. Over half screened positive for a likely stimulant use disorder. (Table 2).

### Qualitative Themes

Across the interviews, we identified and categorized barriers and facilitators to PrEP uptake according to the levels of the social ecological model. Based on this categorization, we identified four major themes: 1) at the individual-level, participants demonstrated high awareness but mixed knowledge and perspectives of PrEP and 2) substance use was interconnected with perceived HIV risk and PrEP interest; 3) at the interpersonal level, participants’ social networks were fragmented and disconnected, with almost no communication of PrEP; and 4) at the structural level, PrEP cost and access were considerable barriers to PrEP uptake among MSM who use stimulants.

### Theme 1: Individual-Level Factors (Awareness, Knowledge, and Perspectives)

The majority (n=18, 85.7%) of participants were aware of PrEP prior to the interview, yet knowledge of PrEP varied widely. Some participants had a basic understanding of how PrEP prevents HIV infection. For example, one participant described PrEP as follows:

Participant 13: I hear it’s preventative to help you from contracting the HIV virus and everything, but I never went into depth or any details, anything at all.

Basic understanding of PrEP was similarly described by another participant:

Participant 18: Well, it reduces the risk of getting infected with HIV. You take the pill Truvada once a day. And it helps prevent the risk of getting infected with HIV. But you have to take, you have to take it daily and have in your system in case you become infected with it.

On the other hand, some participants reported misinformation about PrEP, such as that it protects against sexually transmitted diseases (STDs) other than HIV.

Participant 8: I know that it’s just a pill you can take on a daily basis or before you have sex, that it’s not 100 percent to prevent, but it can help prevent STD or AIDS or something.

Some participants viewed PrEP as a new medication or one still in development. Participant 8 discussed not wanting to start PrEP because: *“ I didn’t really want to experiment, add a whole ‘nother drug into my system, so I refused it.”* At the end of the interview, another participant asked about PrEP:

Participant 21: Where is the research, and where is it in its process?

Incomplete or inaccurate information regarding PrEP resulted in safety concerns. Many participants identified potential side effects as one such concern.

Participant 1: We know this drug, Truvada. I mean, it’s been around. But I don’t know what the long-term effects of it are. I don’t know what would happen if I stopped taking it after being on it. Would I be more susceptible to infection somehow? Is it going to build resistance? Is it going to create more virulent strains of HIV?

Participant 24: Maybe hair loss or something like that, the side effects. I don’t know. Taking a medication like that, what would be the side effects? My hair loss? My liver? Things of that nature.

Some participants expressed similar concerns about medication burden and the potential impact of taking an additional medication, especially if they did not see an added benefit to using PrEP over other HIV risk reduction strategies (see more in Theme 2 below).

Participant 8: Yeah, and the primary reason was I didn’t want to have to do something added to what I already do with the meds, to add an extra pill, if I’m not trying to be sexually active like that.

Other participants expressed concerns about PrEP interactions with alcohol and other drugs.

Participant 24: Can you take it with alcohol? Can you still drink? What happens if you do happen to do drugs once in a blue moon? So those things I would wanna know.

Interestingly, no participants expressed concerns about PrEP adherence.

Participant 7: They [providers] do text me every night… To me, it’s not really any more helpful… Because, I mean, I already take a pill at night, so.

Participant 8: PrEP would be something I would [take], no matter what, every day, it doesn’t matter to me

When asked explicitly if he thought he would have any problems with taking PrEP every day, one participant explicitly denied this as a concern, describing how he would integrate PrEP into his morning routine.

Participant 13: No, shoot, after breakfast, orange juice, let’s go.

Participants offered a variety of suggestions on how to increase PrEP awareness and knowledge among MSM who use stimulants, including advertising PrEP more widely via mainstream media, social media, and dating apps.

Participant 4: Probably just like maybe a commercial or something, like make it more known to people, ‘cause I feel like not a lot of people actually know about it.

One participant emphasized that promoting PrEP via dating apps would be more effective than in physical venues such as bars and bath houses because more people were meeting virtually.

Participant 6: I don’t go to bars anymore, but I’m hearing that the bars are dead. Bathhouses aren’t as busy as they used to be. Everybody would just rather stay home and be on an app. Because, frankly, you can get a man faster than you can get a pizza on an app.

Participants emphasized the importance of sharing facts and statistics demonstrating the effectiveness of PrEP.

Participant 1: I saw these graphics of somebody who takes PrEP is 99 percent protected. I think that would make a big impression on people. Especially like – there’s this perception that condoms are the safest way to prevent HIV infection. So I feel like – I was surprised when I saw that, that it was more effective than condoms.

Participants emphasized that information about PrEP should come from an “accredited source” (*Participant 5*), so that they knew what information to trust.

Participant 11: I kept on getting like contradicting answers and contradicting responses… That’s why I said the Internet is evil. It lies to you and it tells you the truth all the same sentence.

Participants also recommended connecting education on PrEP to other sexual health/HIV prevention services.

Participant 3: You know, I feel there should be some kind of class that teach, maybe like the sex class that they teach should have some info about PrEP.

Participant 4: I would ask them when they get tested, first. Say, “Hey, do you get tested?” and also, “You know, there’s PrEP.” So, yeah, just bringing up testing first.

### Theme 2: Individual-Level Factors (Intersection of Substance Use, Perceived HIV Risk, and PrEP Interest)

Some participants perceived their risk for acquiring HIV as primarily connected to their substance use.

Participant 1: …unpredictable behavior comes definitely with the drugs and the alcohol for sure. Yeah. Since so much of gay hooking up tends to happen in that context of at least drinking. Whether we’re drinking at home and impulsively hook up with somebody, or drinking out at a bar or whatever.

One participant who had recently stopped using drugs connected their likelihood of engaging in HIV risk behaviors to their use of substances.

Participant 7: I don’t have any partner right now. But before I came into treatment, I had a couple partners and I didn’t use no condom or nothing, you know. I really like to use condoms. But and I was using drugs, you know, so I felt that I was at risk, you know what I mean, and I’m probably not that far off from being at risk again, you know what I mean. Because I’ve only got 90 days clean, you know what I mean. So I felt that I should probably just take it, you know… I gave up, like, drugs but I didn’t give up girls, [laughs] you know, so.

For participants in recovery, abstinence from substance use led them to feel more in control of their lives, including their sexual behavior, and to see themselves as less likely to acquire HIV. As such, they identified PrEP as unnecessary for them.

Participant 8: The only thing, if I was still in that addictive mindset of chasing a drug with the sex, then yeah, it [PrEP] would definitely be a major option. But if you change your lifestyle, and you’re not living that type of way, I’m not afraid of myself of doing something that I can’t control, or if I felt like I couldn’t control the urges to have sex unprotected, then I would definitely protect myself.

Some participants expressed a preference for other HIV risk reduction options, such as condoms or serosorting (i.e., selecting sexual partners based on their HIV status), while also acknowledging the limitations of those methods, especially in their ability to use them every time they had sex.

*Participant 25: So if it* [PrEP] *was an option, I would consider it, but I’d rather just try to be with someone who was clean at the start.*

### Theme 3: Interpersonal Factors (PrEP Attitudes and Communication in MSM Social Networks)

Within social networks described by participants, PrEP stigma was not seen as a barrier or concern. Instead, many participants saw PrEP use among potential sex partners as a conscientious choice that would help to alleviate their concerns about HIV.

Participant 3: I feel that people they don’t judge you for being on PrEP. People are more like considering that you’re taking care of yourself kinda thing.

Participant 5: And, if you’re not positive, maybe you should be on it especially if you’re gay or even bisexual having sex with guys. If you’re not using protection, then yeah. Not everybody does, especially if you’re partying. Especially if you’re in that type of environment.

In fact, some participants with HIV suggested that increasing PrEP use was connected to reductions in HIV-related stigma.

Participant 4: Well, it’s friends, but it’s been people that I’ve had sex with as well in the past. And they knew I was positive and they would just say, “Hey, I’m on PrEP, so everything’s cool.”

That said, many participants did not discuss sex, substance use, or HIV prevention strategies like PrEP with the people they considered part of their social network.

*Participant 26: No, I wouldn’t talk with it* [PrEP] *with nobody*.

Participant 8: Just people that come here [community-based organization], people that do sex work or at-risk that come here, that’s the only people I talk to about it.

Some participants expressed distrust of sexual partners and shared they did not discuss HIV status or PrEP use with sexual partners because they did not trust them to tell the truth about their HIV status or PrEP status.

Participant 2: I mean I don’t really ask [about HIV status] because people lie. It’s up to me to protect myself and when I’m using I just don’t care.

Participant 6: So, a lot of my partners claim to be on PrEP. I don’t always believe that… I think, in general these days, a lot of people say that they’re on PrEP but I think… they’re HIV-positive and they just don’t want to say that. So they’d rather just say they’re on PrEP.

Participants also described not having close connections to the people they used drugs with.

Participant 6: People I party with, I don’t spend any other time than when I’m partying with ‘em… They’re just people I party with. Other than that, I don’t have nothing else in common with ‘em.

In many cases, participants reported circumscribed and fragmented social networks, often due to their substance use.

Participant 4: I’ve lost pretty much everybody I loved because of drugs…they would slowly back away out of my life. Some didn’t want to see it [drug use] anymore. Some I hurt or said something mean to because of being on drugs…they just disappear.

Participant 5: The only one in my family that now has anything to do with me is my aunt. The rest of my family doesn’t really want anything to do with me because of my drug use and the things I have done.

Some participants who stopped using drugs intentionally disconnected from individuals in their social network as a strategy for supporting their recovery.

Participant 2: I stay to myself a lot because I don’t like trying to get mixed up with people out here, like my old friends, I used to smoke and do drugs with and stuff, I need to stay away from them, ‘cause that’s why I’m all out of trouble in the past. I don’t need to be around that stuff. I’m trying to stay away.

### Theme 4: Structural Factors (Access as a Barrier)

Participants had varied experience with prior attempts to access PrEP. In addition to the three participants who had taken or were currently taking PrEP, three participants discussed attempting to access PrEP while another participant described his boyfriend’s experience trying to access PrEP. Regardless of prior experience with PrEP access, participants identified potential cost and insurance coverage as major structural barriers to PrEP.

Participant 24: Is this [PrEP] for free? Is this – how much would this cost?… I’d have to find out from my insurance if they’ll cover this.

One participant described how this barrier persisted even with patient assistance programs that subsidize the cost of the medication.

Participant 4: I tried to get my boyfriend on it, but the insurance wouldn’t cover it… they gave him a little red card and that didn’t do it.

Participants also identified consistent access as a barrier, both in starting PrEP as well as continuing with it, as expressed by Participant 1, who did not reschedule a missed appointment to start PrEP, in part, as he explained *“because you also have to come in every three months”.*

## Discussion:

This is among the first studies to evaluate barriers and facilitators to PrEP use among MSM who use stimulants. We found that most participants were aware of PrEP but knowledge was mixed, resulting in concerns about PrEP side effects and drug interactions. As such, there is still a need for accurate, trustworthy and easily accessible information about PrEP. Interest in PrEP was intricately tied to both substance use and HIV risk perception. Despite low levels of reported stigma, engaging social networks may have limited impact on promoting PrEP. Finally, limited access, primarily due to cost and insurance coverage, remained considerable barriers to PrEP uptake. Our results identify potential gaps and targets for future intervention to improve PrEP uptake in this population.

Other studies have found low levels of PrEP awareness among different populations, such as MSM who do not identify as gay, MSM of color, MSM engaging in condomless anal sex, MSM who use stimulants in the southern United States, and persons who inject drugs (74–78). In contrast, participants in this study demonstrated high awareness of PrEP, although PrEP knowledge varied. Our sample was largely recruited from community-based organizations and an STD clinic where PrEP promotion is common, which may account for the discrepancy. That said, PrEP knowledge varied among participants, who were often skeptical of the information they had about PrEP and emphasized the need for trustworthy sources of information.

Concerns about PrEP were the same as those found in many studies about PrEP uptake, and included side effects, potential interactions with medications and drugs, and cost (29–32, 79). Contrary to other studies conducted on this population, participants, including those with experience taking PrEP, did not perceive daily adherence as a barrier to PrEP uptake (80). It is important to note that this study only explored adherence as a potential barrier to PrEP uptake, given that few participants had experience taking PrEP. Also diverging from other studies, PrEP-stigma was not seen as a concern by most participants (81).

Substance use influenced participants’ perspectives on PrEP as a suitable option for HIV prevention. Some participants in early recovery identified as being most at risk for HIV when they were actively using. They considered themselves good candidates for PrEP when they were using drugs, yet they shared they likely would not have taken PrEP at this time, mostly because they did not identify HIV risk as a priority. These participants felt that, in their recovery, they had more control over their substance use and sexual activity, and so did not see themselves as at risk for HIV or preferred to engage in other HIV risk reduction strategies.

These experiences highlight another challenge in increasing PrEP uptake. MSM who are currently using stimulants may see themselves as at higher risk for HIV and yet may not feel ready to take PrEP. Meanwhile, those in early recovery may feel more ready to take PrEP and also less inclined to see it as appropriate for their lower perceived level of HIV risk. Participants described substance use, treatment, and recovery as an iterative cycle, consistent with what has been described in other research (82). In this cyclical process, MSM who use stimulants may experience fluctuating patterns of HIV risk, also termed “seasons of risk” (83, 84). As such, daily PrEP may not be the most appropriate option. Other forms of PrEP in development, such as “PrEP on demand” (i.e., event-drive or 2-1-1 PrEP) or long-acting formulations, may better meet the needs of MSM who use stimulants. Long-acting injectable formulations, in particular, have demonstrated acceptability among other groups of MSM and people who use drugs (85–89). More research is needed to ascertain whether offering such alternative formulations of PrEP would increase uptake in this population. Additionally, this finding around fluctuating patterns of HIV risk and risk perception also reinforces the importance of offering PrEP widely regardless of perceived or current HIV risk, as suggested by other studies (90).

Researchers have previously described social networks of people who inject drugs as conducive to raising awareness about PrEP (91–94). In contrast, most participants in our study described social networks that were fragmented or circumscribed. Even those with more extensive social networks reported they did not discuss their substance use or sexual behavior with others. Almost all said that they did not know the people that they used drugs with and did not discuss topics such as HIV. Moreover, similar to what has been seen in other studies (95, 96), participants expressed mistrust in information received from their peers and a desire to receive PrEP information from “accredited sources”. Latkin et al. highlighted the importance of considering characteristics of social networks, such as density and stability, when designing network-level interventions (97). Our results suggest that interventions tapping into existing social networks may not be effective with this population. Instead, interventions to develop prosocial connections and community supports, such as the Mpowerment Project (98), may be more appropriate. Additionally, consistent with other research (99–101), integrating interventions to promote PrEP within existing points of contact with health and social services (such as substance use treatment programs) could be effective given the desire to receive information from trusted sources. Further investigation is needed to explore how the social networks of MSM who use stimulants differ from those of other MSM and other people who use drugs. Additionally, given the contextual nature of these findings, future research should explore how the social networks of MSM who use stimulants vary by geography and stimulant of choice (e.g., cocaine, crack, crystal methamphetamine).

Participants identified access, especially cost, as a major barrier to PrEP uptake. This is consistent with other studies, which have found these to be significant deterrents to PrEP use (29–31, 33, 35, 36). Future interventions aiming to increase PrEP uptake in this population should be sure to address potential barriers to access.

Lastly, more research is needed to identify alternative ways to intervene to increase PrEP uptake among MSM who use stimulants specifically. The participants in our study suggested several mechanisms for raising knowledge and interest in PrEP among MSM who use stimulants. These suggestions matched those identified in other studies, such as using dating or hookup apps as a means of increasing PrEP knowledge and awareness (61). Participants also suggested raising PrEP awareness and knowledge through billboards, radio and television advertisements, and websites. Participants highlighted the importance of demonstrating the trustworthiness of sources in these advertisements given the wide availability of inaccurate or incomplete information about PrEP.

### Limitations

This study has some limitations. Potential recall and social desirability biases could have influenced participant responses, especially to questions about their attitudes and experiences related to sexual activity and substance use. Participants were largely recruited from community-based organizations. As such, our findings may not be transferrable to other groups of MSM or to MSM who use stimulants in other parts of the country. Nevertheless, this study presents the perspectives of a group of MSM at risk of contracting HIV and provides important implications for potential PrEP interventions.

## Conclusion:

This study examined the potential to develop social network interventions among MSM who use stimulants to promote PrEP uptake. We found that many participants described themselves as isolated and/or did not discuss their substance use or sexual behavior with their social network. Most participants did not know the people they used drugs with well or at all. Our findings suggest that the existing social networks of MSM who use stimulants may not be a suitable point of intervention and that alternative interventions that build social networks and community supports should be considered. Participant responses emphasize the need for more widespread promotion of PrEP through various forms of media, which could address common concerns around side effects and drug interactions. PrEP accessibility, especially cost, continues to serve as a barrier that can be addressed through PrEP navigation, connecting participants to the most convenient providers and to different PrEP coverage options, such as payment assistance programs. Structural interventions to make PrEP more readily accessible to MSM who use stimulants are also needed. Given the interaction of substance use, sexual activity, HIV risk perception, and interest in PrEP, our findings suggest the need to integrate PrEP messaging and intervention into existing points of contact, such as substance use treatment programs, to build interest among those at highest risk.

## Supplementary Material

1792349_Sup_material.

## Figures and Tables

**Table 1-:** Demographic Characteristics of Sample (N = 21)

	Mean (SD) or N (%)
Age	39.3 (10.6)
Race	
White/Caucasian	12 (57.1%)
Black/African-American	6 (28.6%)
Other	3 (14.3%)
Ethnicity	
Hispanic or Latinx	3 (14.3%)
Not Hispanic or Latinx	18 (85.7%)
Sexual Orientation	
Gay	11 (52.4%)
Straight	3 (14.3%)
Bisexual	4 (19.0%)
Other	3 (14.3%)
HIV Status	
HIV-positive	5 (23.8%)
HIV-negative or status unknown	16 (76.2%)
Incarceration History	
Yes	13 (61.9%)
No	8 (38.1%)

**Table 2-:** Sexual Behaviors and Substance Use (N = 21)

	Mean (Range) or N (%)
Number of anal sex partners as bottom (past 3 months)	4.1 (0 – 50)
Number of anal sex partners as top (past 3 months)	4.1 (0 – 45)
Number of vaginal sex partners (past 3 months)	4.0 (0 – 50)
Number of male oral sex partners (past 3 months)	9.1 (0 – 75)
Number of female oral sex partners (past 3 months)	3.7 (0 – 30)
Exchanged sex for drugs, money, etc. (past 3 months)	12 (57.1%)
Ever been sexually assaulted	8 (36.4%)
Ever had a sexually transmitted disease	10 (45.5%)
Usually meet sexual partners at…	
Bar	10 (45.5%)
Bathhouse	3 (13.6%)
Video store	4 (18.2%)
Sex club	2 (9.1%)
Sex parties	3 (13.6%)
Online	10 (45.5%)
Ever injected drugs	9 (40.9%)
Used in the past three months	
Tobacco	17 (81.0%)
Alcohol	16 (76.2%)
Cannabis	12 (57.1%)
Stimulants	13 (61.9%)
Sedatives	5 (23.8%)
Opioids	3 (14.3%)
Likely substance use disorder*	
Tobacco	14 (66.7%)
Alcohol	10 (47.6%)
Cannabis	11 (52.4%)
Stimulants	11 (52.4%)
Sedatives	4 (19.0%)
Opioids	2 (9.5%)

* cut-off for ASSIST-Lite is a score of 2 (out of 3) for all substances except alcohol, which is a score of 3 (out of 4)

## Data Availability

Redacted transcripts available upon request
