# Supplementary material for: Barriers and Facilitators to HIV Pre-Exposure Prophylaxis Uptake among Men Who Have Sex with Men Who Use Stimulants: A Qualitative Study
Source: AIDS Behav. Author manuscript; Available in PMC 2023 Sep 1. (PMC9378498; doi:10.1007/s10461-022-03633-5)
Supplement: 1792349_Sup_material. [file NIHMS1792349-supplement-1792349_Sup_material_.docx]

**Semi-structured interview guide**

**Study Overview, Warm up and Introduction
BEGIN Recording (state date, time, participant number)**

During this interview, we’re interested in learning more about how we can help prevent people from getting HIV by taking a medication called pre-exposure prophylaxis or PrEP. We will also ask you some questions about the people you normally socialize or hang out with, sex, drugs, and the things you do to keep healthy. We will also ask you to complete a couple of brief questionnaires at the end.

This interview is voluntary, and you are not required to answer all of the questions. We expect this interview to last approximately an hour. You may stop the interview at any time. All of your answers will not have your name or other identifying information on it and will be kept in a private, confidential location. Audio-recordings and transcripts of this interview will be destroyed after publication of our findings.

During the interview, we ask that you please turn off your cell phone. Before we begin, what questions or concerns do you have?

**General/Introduction**

First, I’d like to know a little bit about you as a person.

1. Tell me about yourself. Where are you from? How long have you lived here?
   1. How do you spend most of your time (e.g., work, school)? How long have you been in school? Working?
   2. Tell me about some of the things you usually do with your free time.

**Social Network**

Now, I’d like to know a little about the people you normally hang out with.

1. Tell me about the people you spend the most time with.
   1. Who are they? (Close friends? Acquaintances? Co-workers? Family members? Sex partners?)
2. What things do you and your friends normally do together?
   1. Where do you typically hang out and why? What do you normally do for fun?
3. Who do you feel are the most important or influential people you know? What makes them so important or influential to you?
   1. How do these people influence you? How do you influence them?
4. How do you communicate with the people who are most important to you (e.g. text message, phone call, in person)? What do you talk/communicate about most?
   1. How do you talk about sex? HIV? PrEP? Alcohol and drugs?
   2. Where do you get accurate information about your health?
5. Tell me about drug/alcohol use among you and your friends.
   1. Who do you (or did you) do drugs with? Where do you (or did you) generally use? (e.g. at a party, at someone’s house with friends, alone)
   2. How do you get drugs?
   3. Do/did you do drugs with people outside of your friend group?
   4. Do/did your friends and the important people in your life know about your drug use?

**Sexual Health**

Next, I’m going to ask you some questions about your sexual behaviors.

1. Tell me about the last time you had sex. Who was it with? Where was it? How did you meet this person?
2. Tell me about your sexual partners.
   1. Do your partners tend to be men, women or both? Age? Where do you meet them? What role do you usually take on during sex —top, bottom, versatile?
3. How much do the people we discussed earlier know about your sex life? How do you discuss it? What do they think?
4. When you have sex, how concerned are you about HIV or other STDs? What do you do to keep yourself safe and healthy?
   1. How do you discuss safety with your sex partners? Before or after sex? Do you talk about HIV status? PrEP?
   2. What do you think of using condoms during sex? What are the reasons you use or do not use condoms?
   3. What has been your experience with HIV testing? When was the last time you got tested? How about for other STDs?

**Substance Use**

Now I’d like to know a little bit about your use of alcohol and other drugs.

1. How would you describe your drinking in the past year? How often do you drink? How much do you drink when you do?
2. How would you describe your current or prior drug use? What drugs do/did you usually use? How do/did you use them (e.g., ingest, inject, inhale)?
   1. Tell me about your experiences (if any) with injecting drugs.
3. How much do the people we discussed earlier know about your current/past use of drugs? How do you discuss drugs? What do they think?
4. What are some of the reasons you do/did drink alcohol or use other drugs?
   1. Do/did you ever drink or use drugs before or during sex? How does/did being drunk or high influence sex? How does/did it influence the things you do to be safe/healthy?
   2. Do any of the people we discussed earlier ever pressure you to drink or use drugs?
5. How does/did drinking alcohol or using drugs influence your life more generally?
   1. Do/did you ever forget to do things while drinking or using?
6. How do you feel about alcohol or drug treatment programs?
   1. What expectations do you have for such programs?
   2. What have been your experiences with getting treatment for alcohol or drug use? Have you ever gone to one?
      1. If **yes**, what was the experience like? What did you like about it? What do you think could be different?
      2. If **no**, have you ever thought about going to one? What are the reasons that you did not attend such a program? What would you like to know about alcohol or drug treatment beforehand?
      3. Has anyone in your life been influential in your recovery?

**HIV Prevention**

Now we’re going to talk a little about the ways in which you can keep yourselves and others protected from HIV.

1. What is your HIV status?

| **IF HIV-POSITIVE**   1. Tell me about your experience of being diagnosed with HIV. When and where did it take place? 2. How do you think you acquired HIV? Did alcohol or other drugs play a role? 3. What do you remember doing after you found out you were positive? How long did it take you to see a medical provider? 4. What has been your experience taking HIV medications? Are you able to take the medications as prescribed? Have you had any interruptions in treatment? If so, why? Did drugs or alcohol play a role? 5. How often do you talk to others about your HIV status? For what reasons? Who do you tell? 6. What do you do to keep from transmitting HIV to someone else? Is this easy? Difficult? What would make it easier? | **IF HIV-NEGATIVE**   1. What behaviors put someone at risk for acquiring HIV? What do you consider low risk? What do you consider high risk? 2. What do you think your own risk for getting HIV is based on what you have shared? Why? 3. What do you do to keep from acquiring HIV? Is it easy? Difficult? What would make it easier? |
| --- | --- |

**PrEP Intervention**

Now I’m going to ask you some questions about PrEP for preventing HIV.

1. What do you know about PrEP for HIV prevention? Where did you hear this information?

PrEP is a medication that someone can take daily to keep from getting HIV.

**If HIV negative:**

a. Have you ever taken PrEP? Have you ever considered it? If not, do you think it would be a good idea for you?

b. What types of information would be (or were) helpful in deciding whether or not PrEP is right for you? What would convince you (or did convince you) to start PrEP?

c. Would you be willing to discuss your concerns about PrEP with someone else? Who would you ideally want to talk to about your concerns? (e.g., a doctor, nurse, outreach worker, a friend who is currently on PrEP)

d. How frequently would you want to meet? About how much time do you think it would take to answer your questions and address your concerns?

e. In addition to talking to a provider, what other methods of communication would be helpful to learn about PrEP? (e.g., text messages, emails, phone calls)

f. Researchers are working on a form of PrEP that would require an injection every 2-3 months instead of a daily pill. What do you think about this option? Which form (oral versus injectable) would appeal to you more? For what reasons?

**All participants:**

1. Have you ever talked about PrEP with your friends? What was that conversation like? Would you be willing to talk to your friends about the importance of PrEP?
2. Have you ever talked about PrEP with a sexual partner? What was that conversation like?
   1. How would you feel if your sexual partner(s) told you they were taking PrEP?
3. What would be the best way to provide PrEP information to your friends? What do you think is the most important information to include to get other people to consider PrEP?

**Closing Procedures**

Thank you again for your participation today. We really appreciate your assistance. As a reminder, everything that you shared with me today will be kept confidential.

- Is there anything you would like to talk more about or to know about?
- What final questions or concerns do you have?
- Is there any advice that you’d give to someone thinking about whether or not to use PrEP?

**ADMINISTER AUDIT, ASSIST, Risk Behaviors and Demographic Information sheets.**

Thank you again and here is your reimbursement.
